# Determinants of Knowledge, Attitude and Practice towards preventive measures of COVID-19 among adult residencies in Silte zone, Southern Ethiopia

**DOI:** 10.11604/pamj.2022.41.211.33826

**Published:** 2022-03-15

**Authors:** Shemsu Kedir, Mubarek Yesse, Mohammed Muze, Bedru Argaw, Mohammed Dengo, Tajudin Nesre, Musa Jemal, Bahredin Abdella, Faris Hamdala, Awol Saliya, Tofik Mussa, India Kassim, Abdulfeta Kedir, Tewfik Dilebo, Awel Sunkemo, Yesufe Badege, Duretti Ensarmu, Dereje Abebe, Amare Desalegn, Henok Ayelign

**Affiliations:** 1 Department of Public Health, College of Medicine and Health Sciences, Werabe University, Werabe, Ethiopia,; 2 Department of Nursing, College of Medicine and Health Sciences, Werabe University, Werabe, Ethiopia,; 3 Department of Medical Laboratory, College of Medicine and Health Sciences, Werabe University, Werabe, Ethiopia,; 4 Department of Statistics, College of Natural and Computational Science, Werabe University, Werabe, Ethiopia,; 5 Department of Plant Science, College of Agricultural and Natural Resource, Werabe University, Werabe, Ethiopia,; 6 Department of Siltigna, College of Humanity Sciences, Werabe University, Werabe, Ethiopia,; 7 Department of Biology, College of Natural and Computational Science, Werabe University, Werabe, Ethiopia

**Keywords:** COVID-19, knowledge, attitude, Silte zone, practice

## Abstract

**Introduction:**

coronavirus infectious disease-2019 (COVID-19) is currently a global health threat and an international public health emergency. There is a strong need to reinforce community knowledge, attitude and practice (KAP) to control the spread of the virus. The study aimed to identify the determinants of KAP towards preventive measures of COVID-19 among adult residencies.

**Methods:**

a community-based cross-sectional study design was employed in the communities of Silte zone, Southern Ethiopia. A total of 853 participants were selected using multistage stratified sampling technique. We used structured interview administered questionnaire. The KAP level was presented in descriptive and the associated variables conducted in binary logistic regression model.

**Results:**

overall, 81.7% had good knowledge, 78.4% had a positive attitude, and 43.9% had good practices. Being a female (Adjusted Odd Ratio (AOR): 2.3; 95% Confidence Interval (CI): 1.6-3.3), age between “31-40” (AOR: 1.99; 95% CI: 1-3.8) and able to read and write (AOR: 2.6; 95% CI: 1.7-3.7) were significantly associated factors of good knowledge towards COVID-19. Being urban resident (AOR: 1.8; 95% CI: 1.2-2.6) was significantly associated variable with positive attitude towards COVID-19. Being a government employee (AOR: 1.7; 95% CI: 1.1-2.7), able to read and write (AOR: 4.5; 95% CI: 3-6.7) and having good knowledge regarding COVID-19 (AOR: 2.4; 95% CI: 1.6-3.7) were significantly associated factors with good preventive practice towards COVID-19.

**Conclusion:**

alarmingly low preventive practice towards COVID-19 pandemic was indicated. Therefore, health education and promotion programs aimed at mobilizing and improving COVID-19-related practice are urgently needed, especially for those who are illiterate, having rural residency, or generally among underprivileged populations.

## Introduction

In December 2019, WHO declared the SARS-CoV-2 as pandemic, the virus that causes coronavirus disease 2019 (COVID-19), become the newest virus to cause global health fear [1, 2].

The highly contagious characteristics of COVID-19 makes it harsher and more dangerous and causes a high fatality rate and rapid spread of the viruses from China to more than 210 countries around the world, including Ethiopia. Consequently, on March 11, 2020, the World Health Organization (WHO) declared that COVID-19 is a pandemic disease [3]. The majority of cases at the time of writing have been from the US which is closely followed by Italy, Spain, UK and Germany [4].

Following the occurrence of the first COVID-19 case on March 13, 2020, in Ethiopia, the state of emergency was initiated, banning of all public gathering more than four people, greeting by hand shaking are banned, transportation service providers are to reduce passenger loads by 50% and others also included [5]. The battle against COVID-19 is continuing in Ethiopia. To guarantee the final success, people´s adherence to these control measures are essential, which is largely affected by their knowledge, attitudes, and practices (KAP) towards COVID-19 in accordance with KAP theory [6].

Evidence shows that public knowledge is important in tackling the pandemics [6,7]. By assessing public awareness and knowledge about the virus, deeper insights into the existing public perception and practices can be gained, thereby helping to identify attributes that influence the public in adopting healthy practices and responsive behavior [8]. Studies reported that higher levels of information and education were associated with more positive attitude and preventive practice towards COVID-19 [9]. Studies analyzing attitudes and knowledge about COVID-19 concluded that attitude towards government measures related to minimizing the spread of the epidemic was highly associated with the level of knowledge about COVID-19 [10].

Even though there were limited studies conducted in Ethiopia on this specific problem, the findings so far will not be adequate as the country consists of people who have paramount deference in socioeconomic and cultural factors. Since there were limited studies in the country and no scientific evidence of such a study in this study area. Therefore, this study aimed to determine the level of knowledge, attitude, and practice towards preventive measures of COVID-19 and its determinant factors among adult residents in Silte zone Southern Ethiopia, 2020.

## Methods

**Study design and setting:** the community-based cross-sectional study design was conducted from June 1 to July 2020 in Silte zone. Silte zone is one of the zones of the Ethiopian Southern Nations, Nationalities and Peoples Region (SNNPR) and found 172 km away from the capital city, Addis Ababa. The administrative center of the zone is Werabe town. This zone consists of 3 administration towns, 10 rural Woredas and 202 kebeles. Based on the last Census conducted by the central Stastical agency (CSA), in 2018 this zone has estimated population of 1,101,847. About 13% of the total population is urban inhabitants. A total of 221,208 households were counted in this zone. The study was conducted in both urban and rural weredas.

**Study population and eligibility criteria:** all adults living in the zone were source population of this study. All selected adults who fulfil inclusion criteria were the study population. Adults who are 18 years old and above as well as living for six months in the study area were included in the study. Those difficult to hear or critically ill participants were excluded from the study.

**Sample size determination:** it was calculated using single population proportion formula based on the following assumptions; 50% prevalence (P), 95% confidence level, a margin of error of 5% and design effect of 2. By adding 10% nonresponse rate, the final sample size was 853.

**Sampling procedures:** multi-stage stratified sampling technique was employed in rural and urban residents to include study participants in the research. The total of 13 woredas found in the study area were stratified into two strata, urban, and rural, each containing three and ten Weredas, respectively. In the first stage, out of the three urban weredas, one urban wereda and out of ten woredas four were selected using the simple random technique. Then, 30% of kebeles from both urban and rural weredas were selected to keep the generalizability. In the third sampling stage, a proportional systematic sampling technique of every 9th interval (7772HHs/853) was used. If there were more than one respondent in a selected house who is 18 years old and above, one of them was selected by lottery method. If there was no eligible respondent in a selected household during data collection, we re-visited that household for the second time.

**Data collection tools and measurements:** the structured questionnaire was prepared after reviewing published literatures [10-13]. The questionnaire contains items on socio-demographic and economic factors, Knowledge related items, attitude related items and practice related items. The knowledge section comprised of 17 items assessed the nature of the disease, etiology, symptoms, risk group, testing, transmission, treatment and precautions/preventions. Each item was responded as yes, no or I don´t know. The right answer was labelled as 1 while the wrong answer was labeled as 0. Total score ranges from 0-17 and a cut off level was determined based on the mean. Respondents who scored above the mean were considered to have good knowledge. Attitude section comprised of 15 items assessing the attitude of adult residents toward treatment, infection control procedure and information regarding COVID-19. Response of each item was recorded on a 5-point Likert scale as follows strongly agree (5-point), agree (4-point), Undecided (3-point), disagree (2-point), and strongly disagree (1-point). Response points for each item were summed up and scale scores were calculated. The mean score was calculated as a cut-off value and respondents who scored above the mean were considered to have a positive attitude. The practice section included 12 items regarding the use of facemask and practice of other precautionary measures. Each item was responded as yes (1-point) and no (0-point). Practice items total score was calculated by summing up the item (0-12) scores and mean score was calculated as a cut-off value and respondents who scored above mean were indicated as a good practice toward precautionary measures of COVID-19 [10-13].

**Data quality control, processing and analysis:** data quality was assured by caring out careful design, translation and back translation of the questionnaire, appropriate recruitment of data collectors and by giving adequate training and follow-up for data collectors and supervisors. The questionnaire was pre-tested on 5% of adults from areas not included in actual studies and modified before the main study began. The data were checked for completeness and consistency and then coded, entered and stored into the computer using Epi-data software. Then data was exported to SPSS version 20 statistical packages for analysis. Descriptive statistics were calculated for demographic and economic factors, information related and job-related factors. All predictor variables with a p-value < 0.25 were considered as candidate variables for multivariable logistic regression and finally a p-value <0.05 were considered as statistically significant.

**Availability of data and materials:** the minimum data set was attached in supporting information.

**Ethical considerations:** ethical clearance was obtained from Werabe University Research Ethical Review Board with the reference number WRU/RPD/40/2020 before conducting the study. At the time of data collection, written consent was obtained from the participants. Each participant was requested to sign it to certify that he or she had agreed freely to participate in the study. Those not willing to participate were given the right to do so. Confidentiality of responses was also ensured throughout the research process.

## Results

From 853 sampled populations, 836 (98%) of the study population responded to the questionnaire. More than half (51.3%) of the age of study population was between the age of 31-40 years. 499 (59.7%) were male, 548 (65.6%) were married, 356 (42.6%) were farmer and 534 (63.9%) were rural in residency. Thirty per cent of the participants had COVID-19 sources of information from television (Table 1).

**Table 1:** socioeconomic and demographic characteristics of the study participants, Silte zone, Southern Ethiopia, 2020

Variables (n=836)	Category	Frequency	Percent (%)
Sex	Male	499	59.7
	Female	337	40.3
Age (Years)	20-30	254	30.4
	31-40	429	51.3
	41-50	97	11.6
	>50	56	6.7
Religion	Muslim	654	78.2
	Orthodox	128	15.3
	Protestant	54	6.5
Ethnicity	Silte	675	80.7
	Gurage	80	9.6
	Others	81	9.7
Occupation	Farmer	356	42.6
	Merchant	161	19.3
	Govt employee	171	20.5
	Non-employed	148	17.6
Educational status	Not able to read and write	198	23.7
	Able to read and write	638	76.3
Marital status	Married	548	65.6
	Single	240	28.7
	Other	48	5.7
Residency	Urban	302	36.1
	Rural	534	63.9
Monthly income (Ethiopian Birr)	<2,500	246	29.4
	2,500-5,000	298	35.6
	5,000-10,000	212	25.4
	>10,000	80	9.6
Source of information	TV	278	33.3
	Radio	261	31.2
	HCWs	135	16.1
	Friends	162	19.4

### Knowledge of participants regarding COVID-19

Most of the female participants (86.6%) were represents good knowledge. 81.7% (95% CI = 78.9%-84.3%) participants had good knowledge about COVID-19. Sex, age and educational status were candidate variables of knowledge towards COVID-19 for multivariate analysis. Females had 2.3 times more odds of having good knowledge towards COVID-19 when compared to males (AOR: 2.3; 95% CI: 1.6-3.3). Participants who are between “31-40” years old were 1.99 times more likely to have good knowledge towards COVID-19 when compared to the elders (AOR: 1.99; 95% CI: 1-3.8). Moreover, participants who can read and write were had 2.6 times more likely to have good knowledge of COVID-19 when compared to those who were unable to read and write (AOR: 2.6; 95% CI: 1.7-3.7) (Table 2).

**Table 2:** factors associated with knowledge of COVID-19 among adults in Silte zone community, Southern Ethiopia, 2020

Variables (n=836)	Category	Knowledge status		Odds ratio at 95% CI		P-value
		Good (n)	Poor (n)	COR (95% CI)	AOR (95% CI)	
		No. (%)	No. (%)			
**Sex**	Male	251 (74.4)	86 (25.6)	1	1	
	Female	432 (86.6)	67 (13.4)	2.2 (1.5, 3.1)	2.3 (1.6, 3.3)	0.001**
**Age (Years)**	20-30	204 (80.3)	50 (19.7)	1.493	1.46 (0.7, 2.9)	0.274
	31-40	363 (84.6)	66 (15.4)	2.012	1.99 (1.0, 3.8)	0.042*
	41-50	75 (77.3)	22 (22.7)	1.245	1.25 (0.57, 2.7)	0.569
	>50	41 (73.2)	15 (26.8)	1	1	
**Educational status**	Able to read and write	543 (85.1)	95 (14.9)		2.6 (1.7, 3.7)	0.001**
	Not able to read and write	140 (70.7)	58 (29.3)	1	1	

* indicates P<0.05, **indicates P<0.001

### The attitude of participant towards COVID-19

In this study, 633 (75.7%) (CI = 73.1%-78.8%) of participants had demonstrated positive attitude. Sex, place of residency and marital status were candidate variables for multivariable binary logistic regression of attitude towards COVID-19. Urban residents were 1.8 times more likely to have a positive attitude towards COVID-19 when compared to the rural residents (AOR: 1.8; 95% CI: 1.2-2.6) (Table 3).

**Table 3:** factors associated with Attitude towards COVID-19 among adults in Silte zone community, Southern Ethiopia, 2020

Variables (n=836)	Category	Attitude		Odds ratio at 95% CI		P-value
		Positive	Negative	COR (95% CI)	AOR (95% CI)	
		No. (%)	No. (%)			
**Sex**	Male	238 (70.6)	99 (29.4)	1	1	
	Female	395 (79.2)	104 (20.8)	1.5 (1.1, 2.2)	1.3 (0.9, 1.8)	0.097
**Marital status**	Married	416 (75.9)	132 (24.1)	1.5 (0.8, 2.9)	1.1 (0.7, 1.6)	0.15
	Single	185 (77.1)	55 (22.9)	1.6 (0.8, 3.2)	0.7 (0.3, 1.2)	0.13
	Other**	32 (66.7)	16 (33.3)	1	1	
**Residency**	Urban	251 (83.1)	51 (16.9)	1.95 (1.3, 2.8)	1.8 (1.2, 2.6)	0.002*
	Rural	382 (71.5)	152 (28.5)	1	1	

* indicates P<0.05, ** Other: divorced and widowed

### The preventive practice of participants towards COVID-19

Nearly half of (43.9% 95% CI = 40.7%-47%) participants had good preventive practices towards COVID-19. Occupation, educational status, source of COVID-19 information and knowledge of participants regarding COVID-19 were candidate variables of preventive practice towards COVID-19 for multivariable binary logistic regression analysis. Participants who were government employee were 1.7 times more likely to demonstrate good preventive practices towards COVID-19 when compared to non-employed (AOR: 1.7; 95% CI: 1.1-2.7). In addition to this, participants who were able to read and write were 4.5 times more likely to demonstrate good preventive practices towards COVID-19 when compared to those who didn´t read and write (AOR: 4.5; 95% CI: 3-6.7). Finally, participants who had good knowledge about COVID-19 were 2.4 times more likely to demonstrate good preventive practices towards COVID-19 when compared to participants who had poor knowledge towards COVID-19 (AOR: 2.4; 95% CI: 1.6-3.7) (Table 4).

**Table 4:** factors associated with preventive practice towards COVID-19 among adults in Silte zone community, Southern Ethiopia, 2020

Variables	Category	Preventive practices		Odds ratio at 95% CI		P-value
		Good	Poor	COR (95% CI)	AOR (95% CI)	
		No. (%)	No. (%)			
**Occupation**	Farmer	166 (47)	187 (53)	1.6 (1, 2.3)	1.5 (0.9, 2.2)	0.054
	Merchant	68 (42)	94 (58)	1.3 (0.8, 2)	1.37 (0.85, 2.2)	0.19
	Govt-employee	79 (46.7)	90 (53.3)	1.6 (1, 2.5)	1.7 (1.1, 2.7)	0.029**
	Non-employed	54 (35.5)	98 (65.5)	1	1	
**Educational status**	Able to read and write	329 (51.6)	309 (48.4)	4.48 (3, 6.5)	4.5 (3, 6.7)	0.001**
	Not able to read and write	38 (19.2)	160 (80.8)	1	1	
**Source of information**	TV	125 (45)	153 (55)	1.3 (0.8, 1.9)	1.1 (0.7, 1.6)	0.63
	Radio	112 (42.9)	149 (57.1)	1.2 (0.8, 1.8)	1 (0.6, 1.5)	0.87
	HCWs	68 (50.4)	67 (49.6)	1.6 (1, 2.6)	1.4 (0.8, 2.3)	0.17
	Friends	62 (38.3)	100 (61.7)	1	1	
**Knowledge regarding COVID-19**	Good	329 (48.2)	354 (51.8)	2.8 (1.9, 4.1)	2.4 (1.6, 3.7)	0.001**
	Poor	38 (24.8)	115 (75.2)	1	1	

indicates P<0.05, **indicates P<0.001

## Discussion

This study was conducted to identify determinants of knowledge, attitude and practice towards preventive measures of COVID-19 among adult residents in Silte zone, Southern Ethiopia. In this study, 81.7% of participants had good knowledge about COVID-19. This result is concurrent with the studies conducted in Tanzania, Malaysia, Saudi and Nepalese [12,14-16]. However, it is lower than findings reported in Ethiopia, Uganda, Iran and China [13,17-19]. On the other hand, the finding is higher than studies conducted in Ethiopia like Jimma, Mizan-Aman, Dessie and Kombolcha [20-22]. As well, this finding is also higher than the studies conducted in Egypt, Bangladesh, Paraguay and United Arab Emirates (UAE) [10,23,24]. This difference might be related to variations in study area, period and methodological difference. Efforts in battling the pandemic across countries may not be the same and this may lead to deference in knowledge. Another possible reason is that it could be due to differences in the cut-off points used to categorize knowledge. It may also due to differences in the socioeconomic status and the availability of infrastructures. Significantly associated factors of knowledge towards COVID-19 in this study were sex, age and educational status. In this study, females were 2.3 times more likely to have good knowledge about COVID-19 when compared to males. This finding is comparable with the studies conducted in Mizan-Aman [20], Saudi [15], Iran [17] and Tanzania [16]. This is maybe due to that the occurrence of the pandemic disease and its impacts getting the attention of females. In contrast to this finding, the studies conducted in Dessie and Kombolcha [21] revealed that being male was more likely to had good knowledge about COVID-19 compared to females. This might be the coverage of the study area lead to the difference. Participant who were between “31-40” years old were 1.99 times more likely to have good knowledge about COVID-19 when compared to the elders. This finding is in line with the studies conducted in Dessie and Kombolcha [21], Mizan [20] and Egypt [10]. This might be due to the reason that participants who are between “31-40” years old mostly have an access to modern technologies in Ethiopia. Hence, they may have better knowledge about COVID-19 when compared to elders. This study also revealed that participants who were able to read and write were 2.6 times more likely to have good knowledge about COVID-19 when compared to the counterpart. This finding is concur with the studies conducted in Dessie and Kombolcha [21], Mizan-Aman [20] and Iran [17]. This might be due to the reason that participants who are able to read and write may have better access to media which is the one of the most important source of information for acquiring basic knowledge about prevention and control modalities of COVID-19 infections.

The findings of this survey showed that the estimated positive attitude towards COVID-19 was 75.7% (CI = 73.1%-78.8%). This finding is nearly the same as the studies conducted in China 73.8% [13] and UAE 78% [25]. However, it is higher than study reports from Uganda 51.3% [19] and Bangladesh 49% [24]. The discrepancy may be subjected to variation in the cut-values to measure the favorable and unfavorable attitude levels. Besides, the discrepancies may also be due to differences in sample size, study design, population, and study settings. The current study also revealed that urban dwellers were 1.8 times more likely to have positive attitude towards COVID-19 when compared to their counterparts. This finding is comparable with the studies conducted in China [13]. This might be due to the reason that people in urban settings have better access to media and information when compared to their counterparts.

According to this study, 43% of participants showed good practice towards preventive measures towards COVID-19 which relatively low figure. Overall, participants in this study showed partial adoption to the practices recommended to limit the spread of COVID-19. The majority of the participants reported wearing a mask when outside the home, and washing their hands for at least 20 seconds with soap or using hand sanitizer continuously according to WHO recommendations and covering nose and mouth with hand or tissue while sneezing or coughing. However, most of the respondents testified that they were not avoiding crowded places, touching mouth, nose and eyes frequently, greeting relatives and were not maintaining the recommended two meter-distance from other people. In this study, findings revealed that government employee, educational status who are able to read and write and knowledge about COVID-19 were significant associated variables for preventive practices towards COVID-19. This finding concur with the studies conducted in China [13]. These findings clearly indicate the importance of improving residents´ COVID-19 knowledge via health education, which may also result in improvements in their Attitudes and practices towards COVID-19.

Our study has strengths as well as some limitations. We used a cross-sectional survey, which could not assess the changes, causes and effect relationship. Moreover, as we used interviewee administered questionnaire, there might be a possibility of information bias. Despite its few limitations, our study also has strengths. Our study assesses the level of knowledge, attitude and practice towards COVID-19 at the same time. Furthermore, the study included both the rural and the urban settings so that the finding is generalizable to community of the study area. The study would probably provide adequate up-to-date information and improve preventive practices of community of the study area.

## Conclusion

Alarmingly low preventive practice regarding the COVID-19 pandemic was indicated in this study. Therefore, health education programs aimed at mobilizing and improving COVID-19-related practice are urgently needed, especially for those who are illiterate, having rural residency, or generally among underprivileged populations. We suggest public health authorities should attempt and prioritize policies and communication efforts to accommodate the underserved´s needs.

### What is known about this topic

- Coronavirus 2019 is currently a global health threat and an international public health emergency;
- The knowledge and attitudes of the public are expected to largely influence the degree of adherence to the preventive measures and ultimately the clinical outcome;
- Evidence shows that public knowledge is important in tackling the pandemics.

### What this study adds

- Being female had a good attitude towards preventive measures of COVID-19;
- We are determined the magnitude of KAP towards COVID-19 to the study area.
